# Bariatric surgery as a risk factor for alcohol use disorder: a clinical case and literature review

**DOI:** 10.1192/j.eurpsy.2023.824

**Published:** 2023-07-19

**Authors:** J. M. Paupério, F. S. Martins, M. J. Peixoto, M. Mota

**Affiliations:** Centro Hospitalar Universitário de São João, Porto, Portugal

## Abstract

**Introduction:**

Bariatric surgery is a relatively safe surgical procedure with high success rates. The improvement on patient’s self-esteem and overall quality of life are expected benefits that extend beyond physical health. Nonetheless, current literature describes a higher risk of developing alcohol use disorder (AUD) in patients undergoing bariatric surgery that is not better explained by differences in baseline characteristics such as socioeconomic factors. This reinforces the need to optimize mental health professionals’ intervention in these patients.

**Objectives:**

Report a clinical case and conduct a literature review on the etiology of the increased risk of alcohol use disorder in patients after bariatric surgery.

**Methods:**

Description of a clinical case and a non-systematic review of literature on the PubMed database, selecting articles published in the last decade and their included reference lists, combining the following MESH terms: “bariatric surgery” and “abuse, alcohol”.

**Results:**

The clinical case presented is a 45 years old woman, with a college degree, that was diagnosed with AUD in 2020, 6 years after being submitted to bariatric surgery. She didn’t have any alcohol-related problems before the procedure but she describes the death of her mother in 2020 as the trigger for her current heavy drinking habits. Injuries induced by alcohol intoxication have since then caused multiple visits to the emergency room and she has now a reported cognitive impairment that severely compromises her ability for self-care. The association between AUD and bariatric surgery, as presented in the reported clinical case, is well established in current literature. The switch of patient’s coping mechanisms from eating to drinking, surgical-induced alcohol pharmacokinetics changes or adaptations of neuroendocrine mechanisms (such as ghrelin and it’s impact on the mesolimbic dopamine system) that contribute to the augmentation of brain reward signaling are all factors that could play a role in the increased reinforcing value of alcohol in these patients. Psychosocial factors were also identified as variables that could impact alcohol-misuse post-operatively. Furthermore, data suggests differences between surgical procedures: while both Roux-en-Y-Gastric Bypass (RYGB) and Sleeve Gastrectomy (SG) dramatically impact alcohol pharmacokinetics, Laparoscopic Adjustable Gastric Banding (LAGB) does not.

**Conclusions:**

There is a strong possibility that the patient’s drinking habits started as a coping mechanism, but her surgery may have also contributed to her AUD. Our review found multiple biopsychosocial factors that could explain the link between bariatric surgery and AUD and some predictors for its development, but future research is needed to fully elucidate its complexities. Nonetheless, health professionals must be well informed for thetimely prevention, diagnosis and treatment of eventual AUD in these patients.

**Disclosure of Interest:**

None Declared

